# External validation of the MOG-AR score in pediatric myelin oligodendrocyte glycoprotein antibody-associated disease

**DOI:** 10.1080/07853890.2026.2735072

**Published:** 2026-10-19

**Authors:** Sha Xiao, Li Li, Zhimin Zou, Yongjie Liu, Longlong Xie, Kuikui Zeng, Maxuan Hu, Wenliang Yang, Sai Yang, Lingjuan Liu

**Affiliations:** a Department of Pediatrics, The Second Xiangya Hospital, Central South University, Changsha, Hunan, China; b Department of Neurology, The Affiliated Children’s Hospital of Xiangya School of Medicine, Central South University (Hunan Children’s Hospital), Changsha, China; c Department of Radiology, The Affiliated Children’s Hospital of Xiangya School of Medicine, Central South University (Hunan Children’s Hospital), Changsha, China; d Pediatrics Research Institute, Hunan Children’s Hospital, Central South University, Changsha, Hunan, China

**Keywords:** MOG-AR score, myelin oligodendrocyte glycoprotein antibody-associated disease, neurology, pediatric

## Abstract

**Background:**

Predicting the risk of relapse in myelin oligodendrocyte glycoprotein antibody-associated disease is essential for guiding early treatment decisions. A score evaluating onset age, sex, attack phenotype, and treatment received (MOG-AR) has been proposed to identify patients at high relapse risk since disease onset, but has never been validated in a pediatric population. This study aimed to externally validate the MOG-AR score in an exclusively pediatric cohort.

**Patients and methods:**

We retrospectively identified 139 children with myelin oligodendrocyte glycoprotein antibody-associated disease (age <18 years) within the past decade. The MOG-AR score was calculated and the patients were stratified into four risk grades. Discrimination and calibration were assessed using multiple complementary metrics, and univariable analyses were performed for each score component. Sensitivity analyses included exploratory reclassification using modified MOG-AR grade thresholds and the development of an exploratory phenotype-based model.

**Results:**

We included 139 children with a median follow-up of 43 months; relapse occurred in 28.8% of patients. Harrell’s concordance index was 0.472 (95% confidence interval, 0.366–0.577), and the time-dependent area under the receiver operating characteristic curve ranged from 0.467 to 0.510 over 1–5 years. Calibration assessment indicated limited variation in predicted probabilities across risk grades. Modified MOG-AR grade thresholds did not improve risk group separation (log-rank *p* = 0.359) or alter continuous scores or rankings. The exploratory phenotype-based model showed group separation on log-rank testing (*p* = 0.031) but poor discrimination (C-index, 0.394).

**Conclusions:**

In this single-center pediatric cohort, the MOG-AR score did not provide clinically useful stratification of first-relapse risk. Limited variability in the age and treatment-related components may have contributed to its limited performance. Further multicenter validation in pediatric cohorts with broader case-mix and treatment patterns is warranted. The exploratory phenotype-based model requires independent prospective validation.

## KEY MESSAGES

- In this exclusively pediatric cohort, the MOG-AR score showed limited discrimination for time to first relapse (C-index, 0.472; 1–5-year time-dependent AUCs, 0.467–0.510).
- The age component was noncontributory, and the treatment-related components showed restricted variability, restricting evaluation across the score’s intended range of predictors and risk.
- These findings are specific to this single-center cohort and support further multicenter validation in pediatric populations with broader case-mix and treatment patterns before the score’s general applicability to pediatric populations can be established.

## Introduction

Myelin oligodendrocyte glycoprotein antibody-associated disease (MOGAD) is a recently recognized distinct central nervous system demyelinating disorder [1,2]. Relapses occur in 40–60% of cases [3,4], increasing to approximately 70% over follow-up periods exceeding 5 years [5,6]. Repeated attacks drive cumulative neurological disability [7], and early identification of high-risk patients is essential to guide the timely initiation of maintenance immunotherapy [1,8,9]. However, reliable tools for predicting the risk of relapse from disease onset are lacking. Several factors have been associated with the relapse risk, including MOG-IgG antibody persistence, age, sex, onset phenotype, early treatment with immunosuppressive agents, and prolonged corticosteroid use [4,9–16].

A score evaluating onset age, sex, attack phenotype, and treatment received (the MOG-AR score) has been proposed to identify patients at high risk since disease onset [10]. External validations have yielded inconsistent results. Acceptable discrimination was confirmed in an independent Chinese cohort [17], whereas suboptimal performance was reported in both a large UK cohort and an Italian multicenter study [17,18]. However, to our knowledge, no external validation of the MOG-AR score has been conducted in pediatric cohorts.

MOGAD differs between children and adults in its clinical phenotype, disease course, and treatment. It occurs relatively more frequently in children and represents an important cause of acquired CNS demyelination in childhood [19]. Younger children with MOGAD commonly present with acute disseminated encephalomyelitis (ADEM) [20,21]. Maintenance immunotherapy after a first MOGAD attack remains individualized in both adults and children, with treatment decisions guided by relapse risk, disease phenotype, and treatment-related considerations [13,22]. These age-related differences raise questions about the applicability of a score developed in mixed-age cohorts to pediatric populations. Therefore, we externally validated the MOG-AR score in an exclusively pediatric MOGAD cohort, evaluated its discrimination and calibration, and conducted sensitivity and exploratory analyses to characterize its performance in this setting.

## Patients and methods

### Study design and patient inclusion

This single-center retrospective cohort study was conducted at the Affiliated Children’s Hospital of Xiangya School of Medicine, Central South University (Hunan Children’s Hospital), between September 2015 and July 2025. The study was approved by the Ethics Committee of the Affiliated Children’s Hospital of Xiangya School of Medicine, Central South University (Hunan Children’s Hospital) [Approval No. KYSQ2025-252] and was performed in accordance with the ethical standards of the Declaration of Helsinki. Informed consent was waived owing to the retrospective study design; all procedures were part of routine clinical care.

The inclusion criteria were: (1) fulfillment of the 2023 International MOGAD Panel proposed diagnostic criteria [1]; (2) onset age below 18 years; (3) complete clinical records and treatment history with dates for the first acute attack; (4) complete serum or cerebrospinal fluid (CSF) MOG-IgG testing results; and (5) relapsing disease course or monophasic disease with at least 6 months of follow-up. The exclusion criteria were: (1) incomplete treatment records and (2) loss to follow-up or critical missing data precluding analysis. Of the 156 screened patients, 139 were included (Figure 1).

**Figure 1.**
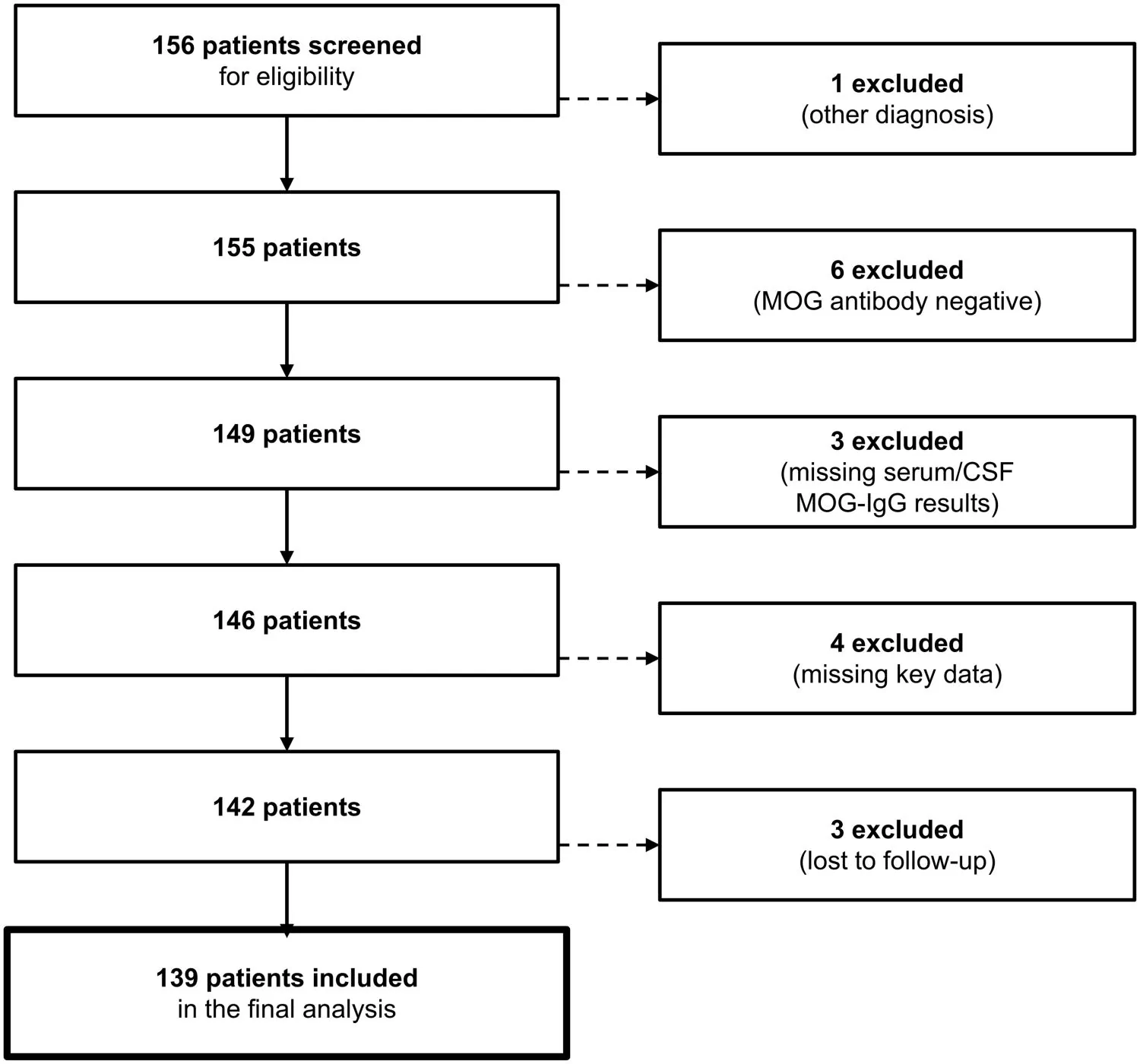
Flow chart of patient enrollment.

### Data collection

Data were extracted through retrospective review of electronic medical records. The collected variables included demographic information (sex and age at onset), date of MOGAD onset, attack phenotype at first presentation, acute treatment received, maintenance immunotherapy use, laboratory findings (complete blood count, C-reactive protein level, CSF routine examination, and biochemistry), disease course and relapse events during follow-up, and Expanded Disability Status Scale score at the last follow-up. The attack phenotypes were classified according to the predominant clinical and radiological syndromes at the first attack: optic neuritis, transverse myelitis, ADEM, cerebral cortical encephalitis, brainstem or cerebellar deficits, and cerebral monofocal or polyfocal deficits.

Serum MOG-IgG was detected using a live cell-based assay. Based on the 2023 International MOGAD Panel proposed diagnostic criteria [1], MOG-IgG titers were dichotomized as clear-positive (titer ≥1:100; values: 1:100, 1:320, 1:1000, 1:3200) and low-positive (titer <1:100; values: 1:10, 1:32). For continuous analyses, MOG-IgG titers were log-transformed prior to inclusion in the regression models to account for the right-skewed distribution inherent to serially diluted antibody measurements.

Relapse was defined as a new clinical attack occurring at least 30 days after the onset of the preceding attack and, when MRI was available, supported by new or enlarging T2/FLAIR-hyperintense lesions and/or new contrast-enhancing lesions corresponding to the clinical syndrome. A monophasic disease course was defined as the absence of relapse during follow-up. Total follow-up duration was calculated from the first MOGAD attack to the last documented clinical assessment for all participants. For time-to-first relapse analyses, time zero was the first MOGAD attack. For patients who relapsed, the date of the first relapse was considered the event date, whereas patients without relapse were censored at their last documented clinical assessment.

### MOG-AR score calculation

The MOG-AR score was calculated for each patient using the original scoring algorithm described by Xu et al. [10] The score integrates five clinical predictors assessed at the time of first attack, with each assigned a weighted point value: onset age ≥45 years (2 points), female sex (2 points), attack phenotype (cerebral cortical encephalitis, 4 points; ADEM, 3 points; optic neuritis, 2 points; cerebral monofocal or polyfocal deficits, 1 point; brainstem or cerebellar deficits, 1 point; myelitis, 0 points (phenotype categories are mutually exclusive and the highest applicable score is assigned); absence of immunosuppressive therapy (IST) use, 5 points; and oral corticosteroid (OCS) duration less than 3 months, 3 points). Total scores range from 0 to 16. Patients were stratified into four risk grades: grade 1 (0–4 points), grade 2 (5–8 points), grade 3 (9–12 points), and grade 4 (13–16 points).

IST use was defined as the receipt of any disease-modifying immunosuppressive agent (including rituximab, mycophenolate mofetil, azathioprine, or tocilizumab) for at least 6 months after the first attack or regular maintenance immunosuppressant use until the next attack or follow-up.

### Sensitivity analysis using modified grade thresholds and exploratory pediatric phenotype model

As an exploratory sensitivity analysis, the original MOG-AR grade thresholds were shifted downward by two points (grade 1, 0–2; grade 2, 3–6; grade 3, 7–10; grade 4, 11–14), while the published scoring algorithm was retained. Because the age component contributed zero points in all participants, this threshold reclassification changed only category assignments and did not change continuous scores or patient rankings. It was not intended as model updating. An exploratory phenotype-based model was additionally constructed because onset phenotype was the only MOG-AR component with meaningful distributional variability in this cohort. Patients were classified into three risk groups: high risk (cerebral cortical encephalitis, optic neuritis, or brainstem/cerebellar deficits without concurrent ADEM or cerebral monofocal/polyfocal deficits), low risk (ADEM or cerebral monofocal/polyfocal deficits without high-risk phenotypes), and intermediate risk (all remaining patients). Discrimination and Kaplan–Meier analyses were performed as described below. This exploratory model was derived and evaluated in the same cohort and has not been externally validated.

### Statistical analysis

Categorical variables are reported as frequency (percentage) and were compared using the χ^2^ test or Fisher’s exact test. Continuous variables are reported as median (interquartile range) and were compared using the Mann–Whitney *U* test or independent-samples *t* test after assessment of normality using the Shapiro–Wilk test. Multiple group comparisons were performed using analysis of variance or the Kruskal–Wallis test.

The present evaluation focused on the performance of the published MOG-AR score for predicting time from the first MOGAD attack to first relapse. We assessed discrimination and calibration. Discrimination was quantified using Harrell’s concordance index (C-index) and time-dependent areas under the receiver operating characteristic curve (AUCs) at 1, 2, 3, 4, and 5 years (time ROC package in R; 95% confidence intervals [CIs] estimated using the influence-function approach).

Multiple indicators were used to assess calibration. Calibration plots were generated to compare predicted and observed survival estimates using bootstrap confidence intervals. The grade 3 and grade 4 subgroups were combined because of the limited sample size. Brier and integrated Brier scores were calculated as measures of overall predictive performance. The observed-to-expected (O/E) ratio was computed with bootstrap confidence intervals, and calibration-in-the-large was defined as the natural logarithm of the O/E ratio. The calibration slope, which reflects dispersion of predicted risks, was estimated using logistic regression [23,24].

Univariable log-rank analyses were performed for the individual MOG-AR score components. Kaplan–Meier curves were generated for MOG-AR grade, post-attack IST use, combined acute-phase treatment (intravenous methylprednisolone alone versus intravenous methylprednisolone with intravenous immunoglobulin and/or plasma exchange), and OCS duration (<3 versus ≥3 months). Curves were compared using the log-rank test.

A two-tailed *p* value of less than 0.05 was considered statistically significant. Statistical analyses were performed using IBM SPSS Statistics version 25.0 for Windows (IBM Corp., Armonk, NY, USA) and R version 2024.12.1.

## Results

### Patient enrollment and baseline characteristics

A total of 139 children with MOGAD were included in the final analysis. The cohort was exclusively pediatric, with onset ages ranging from 13 to 169 months (median [interquartile range] age at onset, 82 [59–121] months; 83 female [59.7%]; 56 male [40.3%]); 99 (71.2%) had a monophasic disease course, and 40 (28.8%) experienced relapse. The baseline characteristics of the patients are presented in Table 1. The monophasic and relapsing groups had similar sex distributions (*p* = 0.67), ages at onset (*p* = 0.76), onset phenotypes, and MOG-IgG titers (*p* = 0.48). Follow-up duration was longer in the relapsing group (46.5 versus 39 months; *p* = 0.02).

**Table 1.** Baseline demographic and clinical characteristics of 139 children with MOGAD, stratified by disease course.

Characteristic	Total (*n* = 139)	Monophasic (*n* = 99)	Relapsing (*n* = 40)	*p* value
**Demographics**				
Female sex	83/139 (59.7)	58/99 (58.6)	25/40 (62.5)	0.67
Age at onset, median (IQR), months	82 (59–121)	82 (53–121)	79 (67–110.8)	0.76
Age ≥72 months, no. (%)	84/139 (60.4)	60/99 (60.6)	24/40 (60.0)	0.95
**Onset phenotype, no. (%)**				
Optic neuritis	20/139 (14.4)	12/99 (12.1)	8/40 (20.0)	0.23
Myelitis	6/139 (4.3)	5/99 (5.1)	1/40 (2.5)	0.67
ADEM	51/139 (36.7)	39/99 (39.4)	12/40 (30)	0.30
Cerebral cortical encephalitis	22/139 (15.8)	14/99 (14.1)	8/40 (20.0)	0.39
Brainstem or cerebellar deficits	7/139 (5.0)	3/99 (3.0)	4/40 (10.0)	0.11
Cerebral monofocal or polyfocal deficits	24/139 (17.3)	20/99 (20.2)	4/40 (10.0)	0.15
Mix	9/139 (6.5)	6/99 (6.1)	3/40 (7.5)	0.76
**CSF findings**				
CSF WBC, median (IQR), ×10⁶/L	30 (8–84)	30 (8–82.5)	30 (11.5–85.5)	0.53
CSF WBC >15 cells/µL, no. (%)	89/139 (65.9)	61/99 (62.9)	28/40 (73.7)	0.23
CSF protein, median (IQR), g/L	0.3 (0.2–0.4)	0.3 (0.2–0.5)	0.3 (0.2–0.4)	0.30
CSF protein >0.5 g/L, no. (%)	26/139 (19.5)	20/99 (20.8)	6/40 (16.2)	0.55
Serum WBC (IQR), ×10⁹/L	12.0 (8.9–18.0)	11.9 (8.5–18)	13.4 (10.7–17.2)	0.23
Serum CRP (IQR), mg/L	0.5 (0.5–6.3)	0.5 (0.5–5.5)	0.5 (0.5–10)	0.67
**MOG antibody**				
MOG-IgG titer, median (IQR), log₁。	2 (2–3)	2 (2–3)	2 (1–3)	0.48
Clear-positive MOG-IgG titer (≥1:100), no. (%)	74/127 (58.3)	57/93 (61.3)	17/34 (50.0)	0.25
**Treatment**				
IVMP alone, *n* (%)	33/139 (23.7)	20/99 (20.2)	13/40 (32.5)	0.12
IVMP + IVIG/plasma exchange, *n* (%)	98/139 (70.5)	72/99 (72.7)	26/40 (65.0)	0.37
Immunosuppressive therapy (IST) use, *n* (%)	7/139 (5.0)	5/99 (5.1)	2/40 (5.0)	0.68
ICU admission, no. (%)	26/139 (19.0)	19/99 (19.2)	7/40 (18.4)	0.92
Oral corticosteroid taper duration, median (IQR), months	6 (3–7)	6 (4–6)	6 (3–9)	0.45
Oral corticosteroid duration ≥3 months, no. (%)	134/139 (96.4)	96/99 (97.0)	38/40 (95.0)	0.63
NSIS maintenance, no. (%)	13/139 (9.4)	11/99 (11.1)	2/40 (5.0)	0.35
**Prognosis**				
Follow-up duration, median (IQR), months	43 (20–65)	39 (16–63)	46.5 (31.5–74.3)	0.02
ARR, median (range)	0.0 (0.0–0.0)	0	0.38 (0.21–0.76)	NA
EDSS ≥1 at last follow-up, no. (%)	14/139 (10.1)	10/99 (10.1)	4/40 (10.0)	>.99

Abbreviations: ADEM, acute disseminated encephalomyelitis; MOGAD, myelin oligodendrocyte glycoprotein antibody–associated disease; MOG-IgG, anti–myelin oligodendrocyte glycoprotein immunoglobulin G; IQR, interquartile range; ON, optic neuritis; EDSS, Expanded Disability Status Scale; IVIG, intravenous immunoglobulin; PLEX, plasma exchange; CSF, cerebrospinal fluid; CRP, C-reactive protein; IVMP, intravenous methylprednisolone; IST, immunosuppressive therapy; ICU, intensive care unit; ARR, annualized relapse rate; NSIS, nonsteroidal immunosuppressant.

### MOG-AR score distribution

The MOG-AR scores ranged from 2 to 13 points (median, 8; mean, 8.4; Figure 2). The age criterion (onset age ≥45 years) contributed zero points to all 139 patients by definition. Most patients had grade 2 (*n* = 70, 50.4%) or grade 3 (*n* = 61, 43.9%), with a few cases of grade 1 (*n* = 6, 4.3%) and grade 4 (*n* = 2, 1.4%). IST was withheld in 132 patients (95.0%), and OCS was administered for ≥3 months in 134 patients (96.4%).

**Figure 2.**
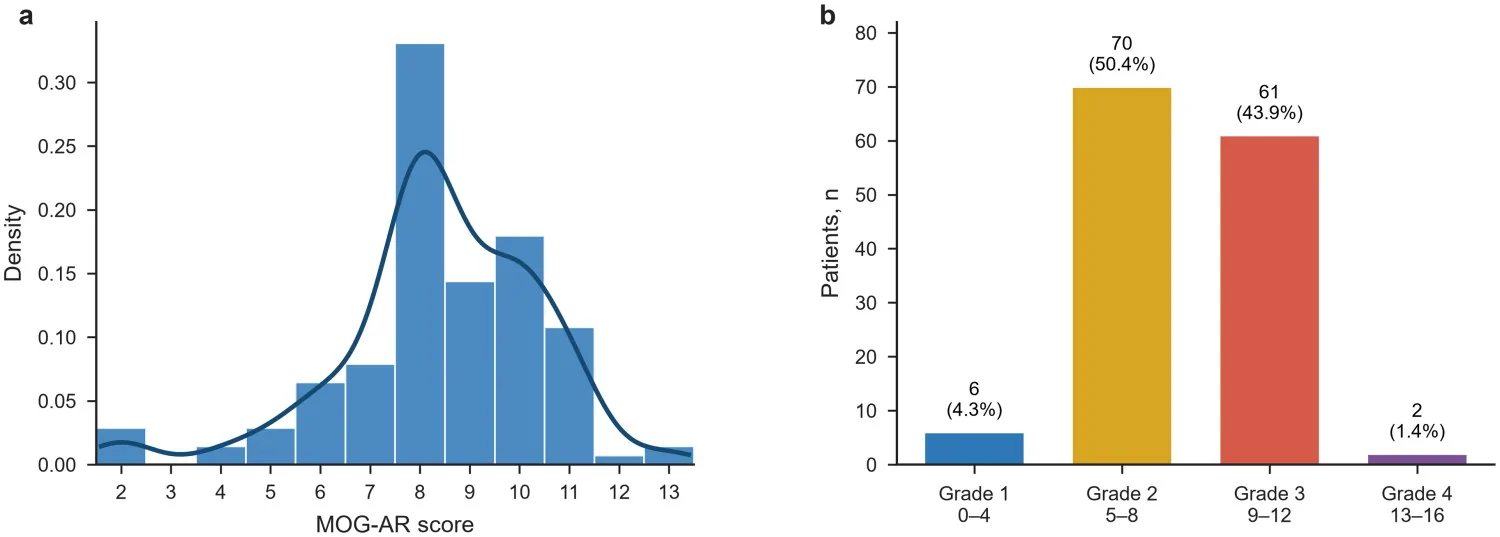
Distribution of MOG-AR scores and risk grades. (A) Histogram and density plot of MOG-AR total scores. (B) Bar chart showing the proportion of patients in each risk grade.

### Predictive performance of the MOG-AR score

The discrimination metrics for the external validation are summarized in Table 2. Harrell’s C-index and time-dependent AUCs at 1, 3 and 5 years were calculated for the MOG-AR score to assess its ability to distinguish patients who experienced relapse from those who did not (Figure 3). The C-index for the MOG-AR score was 0.472 (95% CI 0.366–0.577). Time-dependent AUC values were 0.467 (95% CI 0.336–0.599) at 1 year, 0.491 (0.368–0.614) at 2 years, 0.494 (0.365–0.623) at 3 years, 0.472 (0.343–0.601) at 4 years, and 0.510 (0.374–0.646) at 5 years. Confidence intervals included 0.5 at all time horizons (Table 2).

**Figure 3.**
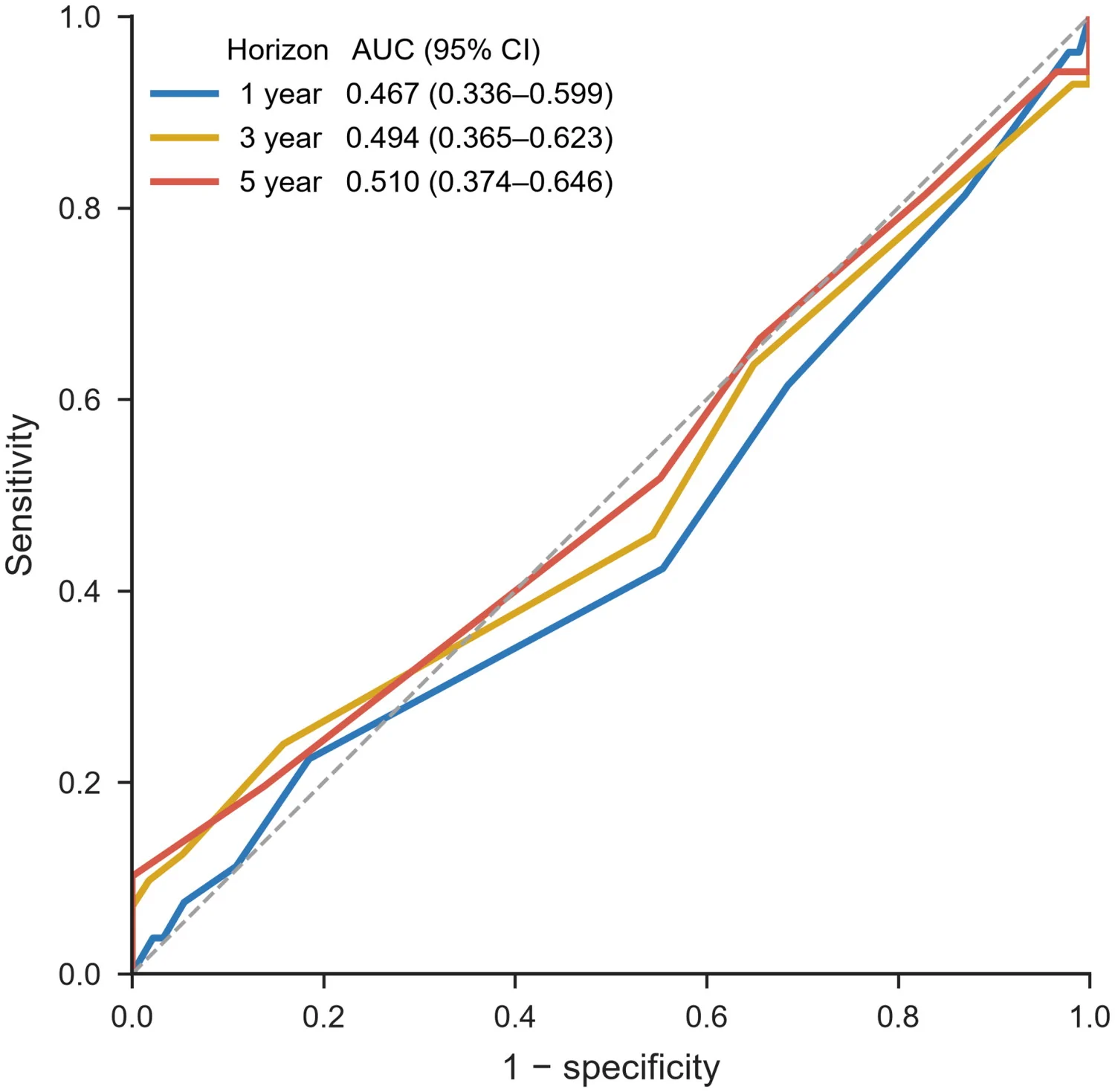
Time-dependent area under the curves of the MOG-AR score.

**Table 2.** Summary of discrimination and calibration metrics for the MOG-AR score in this pediatric cohort.

Measure	Result (95% CI)
C-index	0.472 (0.366–0.577)
Time-dependent AUC, 1 year	0.467 (0.336–0.599)
Time-dependent AUC, 2 years	0.491 (0.369–0.613)
Time-dependent AUC, 3 years	0.494 (0.365–0.623)
Time-dependent AUC, 4 years	0.472 (0.343–0.601)
Time-dependent AUC, 5 years	0.510 (0.374–0.646)
Brier score (36 months)	0.113
Integrated Brier score	0.113
O/E ratio, 1 year	1.004 (0.665–1.321)
O/E ratio, 2 years	1.005 (0.7–1.317)
O/E ratio, 3 years	1.005 (0.677–1.298)
Calibration-in-the-large	0.004 (−0.408–0.278)
Calibration slope	5.492 (−7.164–19.401)

CI, confidence interval.

The Brier score at 36 months was 0.113. The O/E ratio was 1.004 (95% CI, 0.665–1.321) at 1 year, 1.005 (0.700–1.317) at 2 years, and 1.005 (0.677–1.298) at 3 years. All values were close to 1.0, and the confidence intervals included 1.0 at every time point. Calibration-in-the-large was 0.004 (95% CI, −0.408 to 0.278) (Table 2).

We also evaluated the score’s calibration using a plot of predicted versus observed relapse probabilities, which failed to show adequate agreement across all three risk groups (grades 1, 2, and 3/4) (Figure 4). The calibration slope was 5.49 (95% CI −7.16 to 19.40); the CI was wide and included 1.0 (Table 2).

**Figure 4.**
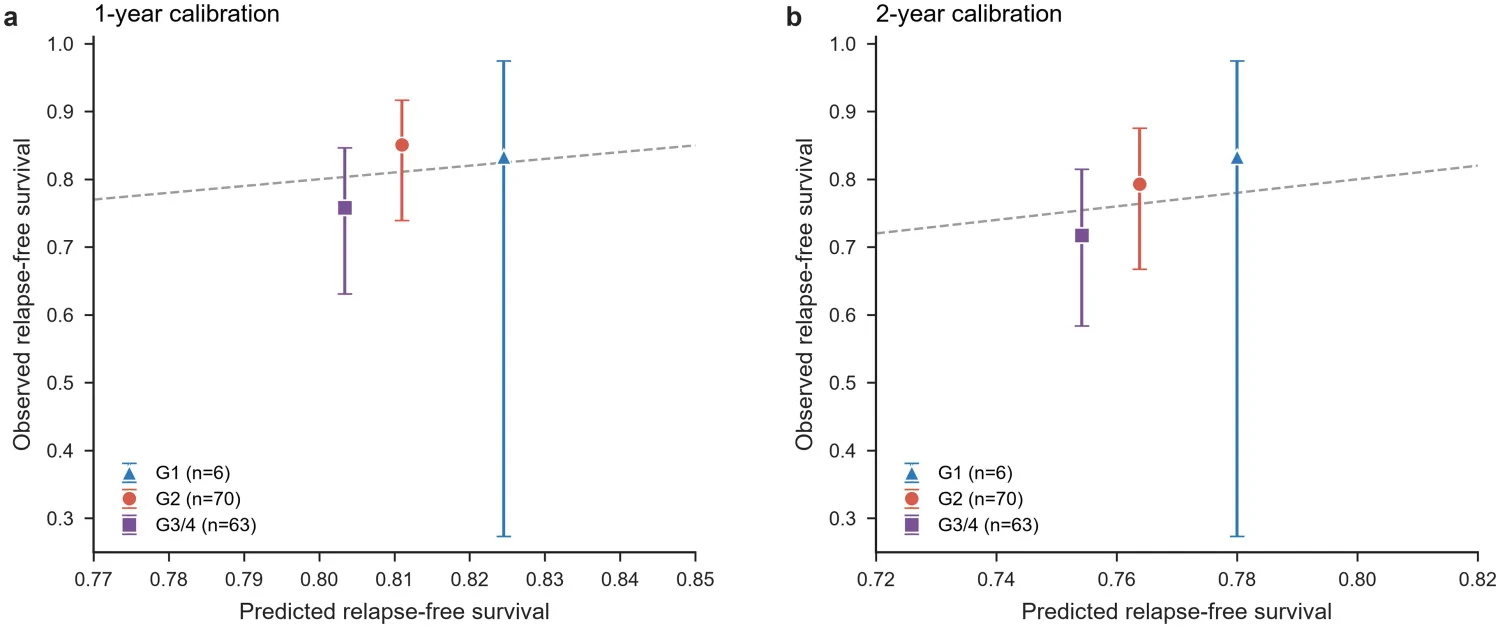
Calibration plots for the MOG-AR score at 1 and 2 years. Error bars represent 95% confidence intervals.

### Univariable analysis and Kaplan–Meier survival analysis

In the univariable log-rank analyses, no individual MOG-AR score component was significantly associated with time to first relapse (Table 3). The age component could not be evaluated because it contributed zero points for all 139 patients. Sex (*p* = 0.969), attack phenotype categories (all *p* > 0.1), non-use of IST (*p* = 0.626), and OCS duration (*p* = 0.843) were not statistically significant.

**Table 3.** Univariable log-rank analysis of each MOG-AR score component against time to first relapse.

Variable	*n* (present)	*n* (relapsed)	*p* value
Female	83	25	0.969
ON	26	10	0.212
Myelitis	6	1	0.467
ADEM	51	12	0.240
Cerebral cortical encephalitis	22	8	0.233
Brainstem or cerebellar deficits	9	5	0.116
Cerebral monofocal or polyfocal deficits	25	4	0.166
IST not use	132	38	0.626
OCS <3 months	5	2	0.843

ADEM, acute disseminated encephalomyelitis; ON, optic neuritis; IST, immunosuppressive therapy; OCS, oral corticosteroid.

Kaplan–Meier analysis demonstrated differences in relapse-free survival across MOG-AR grades (log-rank *p* = 0.032; Figure 5A). Grade 4 (*n* = 2) patients had a 1-year cumulative relapse rate of 50.0% (95% CI 0–87.5%). Among grade 1, 2, and 3 patients, the 1-year cumulative relapse rates were 16.7%, 14.9%, and 23.3%, respectively (Table 4).

**Figure 5.**
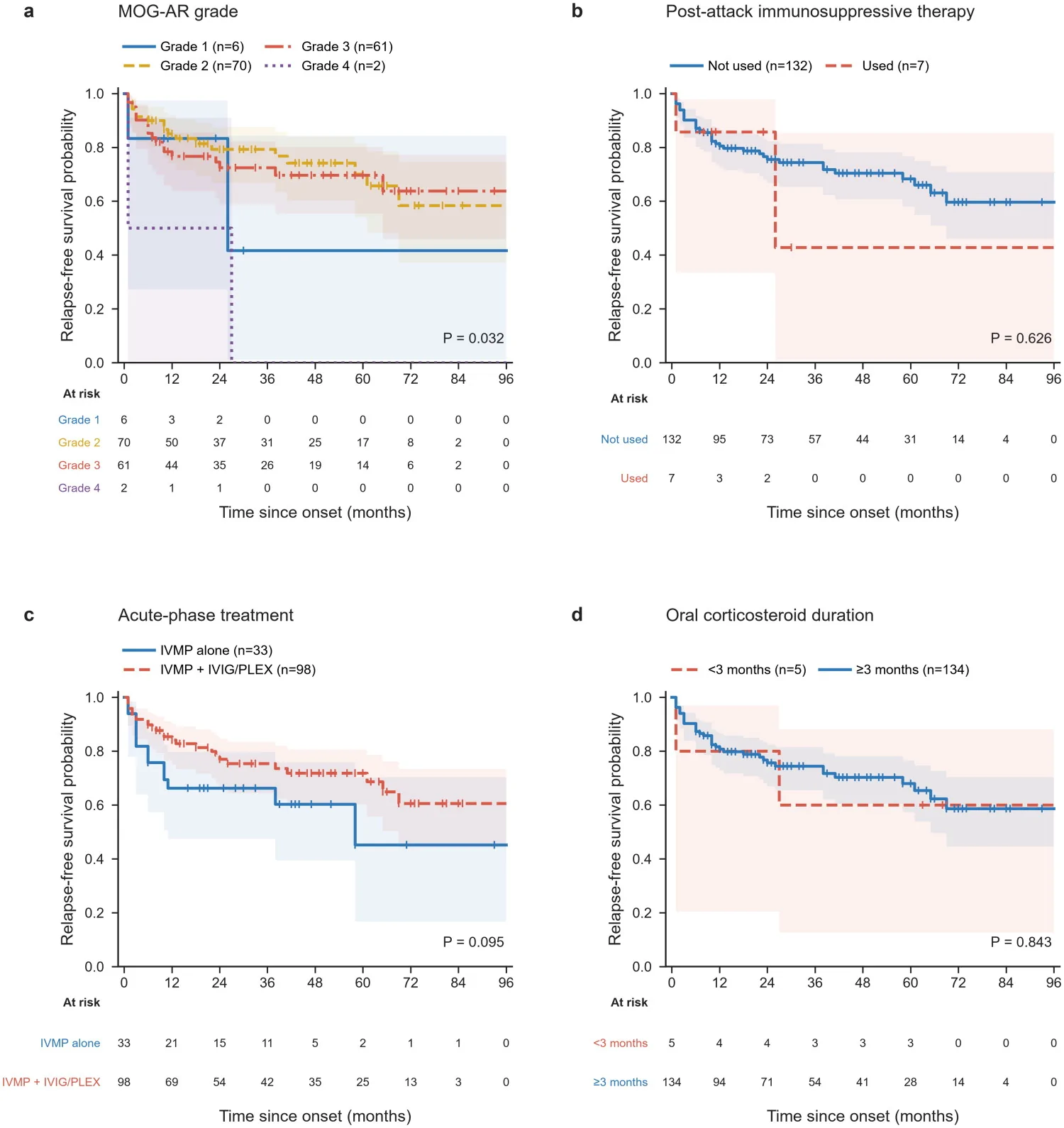
Kaplan–Meier curves for relapse-free survival. (A) Stratified by MOG-AR risk grade. (B) Stratified by post-attack immunosuppressive therapy use. (C) Stratified by combined acute-phase treatment. (D) Stratified by oral corticosteroid duration (<3 versus ≥3 months).

**Table 4.** One-year cumulative relapse risk according to the original MOG-AR grades.

MOG-AR Grade	Score range	Total patients	Patients with relapse	1-year relapse rate, % (95 % CI)
Grade 1	0–4	6	2	16.7 (0–41.7)
Grade 2	5–8	70	18	14.9 (5.9–23.1)
Grade 3	9–12	61	18	23.3 (11.8–33.3)
Grade 4	13–16	2	2	50 (0–87.5)

CI, confidence interval.

Kaplan–Meier analyses showed no significant differences in time to first relapse according to IST use (log-rank *p* = 0.63; Figure 5B), combined acute-phase treatment (log-rank *p* = 0.29; Figure 5C), or OCS duration (<3 versus ≥3 months; log-rank *p* = 0.843; Figure 5D). Only five patients received OCS for <3 months; therefore, this comparison should be interpreted cautiously.

### Sensitivity analysis using modified MOG-AR grade thresholds

Using modified MOG-AR grade thresholds, four patients (2.9%) were classified as grade 1, 15 (10.8%) as grade 2, 102 (73.4%) as grade 3, and 18 (12.9%) as grade 4 (Table 5). One-year cumulative relapse rates were 25.0% (95% CI, 0.0%–57.4%), 13.3% (0.0%–28.9%), 18.3% (10.3%–25.7%), and 27.8% (3.8%–45.8%) for grades 1 through 4, respectively. This reclassification changed category assignment but did not change continuous MOG-AR scores or patient rankings. Risk group separation did not improve (log-rank *p* = 0.359; Figure 6).

**Figure 6.**
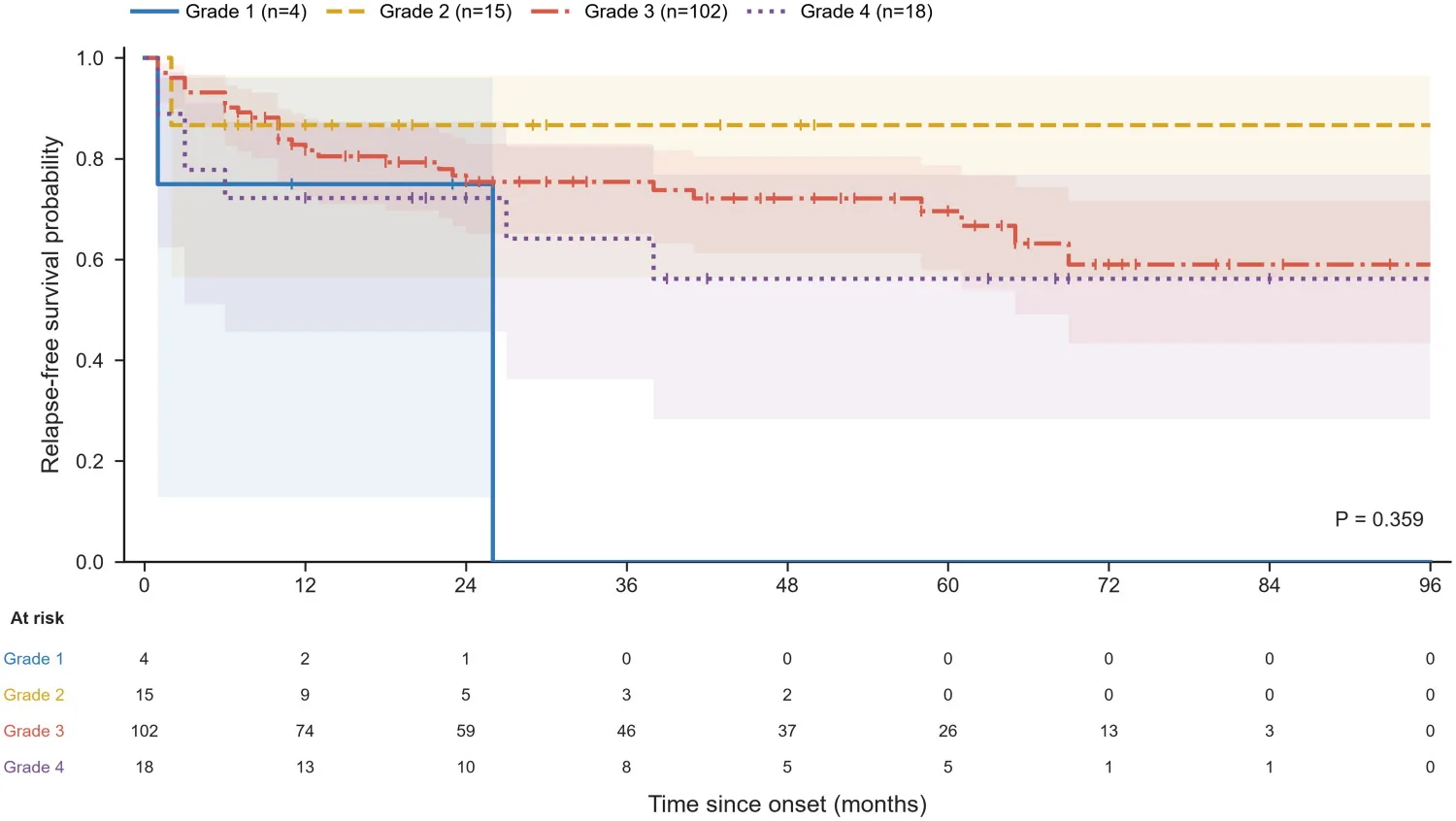
Kaplan–Meier curves for relapse-free survival using modified MOG-AR grade thresholds in the sensitivity analysis.

**Table 5.** One-year cumulative relapse risk using modified MOG-AR grade thresholds (sensitivity analysis).

MOG-AR grade using modified thresholds	Score range	Total patients	Patients with relapse	1-year relapse rate, % (95% CI)
Grade 1	0–2	4	2	25 (0–57.4)
Grade 2	3–6	15	2	13.3 (0–28.9)
Grade 3	7–10	102	29	18.3 (10.3–25.7)
Grade 4	11–14	18	7	27.8 (3.8–45.8)

CI, confidence interval.

### Exploratory pediatric phenotype model

Onset phenotype was the only MOG-AR component with meaningful distributional variability in this cohort. An exploratory phenotype-based model was therefore constructed to assess whether phenotype-derived stratification could generate a hypothesis-driving signal.

Patients were classified into high-risk (*n* = 57, 41.0%), low-risk (*n* = 76, 54.7%), and intermediate-risk (*n* = 6, 4.3%) groups. Overall relapse rates were 40.4%, 21.1%, and 16.7%, respectively. The C-index was 0.394 (95% CI, 0.312–0.475). Kaplan-Meier analysis showed group separation (log-rank *p* = 0.031; Figure 7), with 1-year cumulative relapse rates of 14.0%, 16.7%, and 26.6% in the low-, intermediate-, and high-risk groups, respectively (Table 6). Comparative performance metrics are summarized in Table 7, and time-dependent AUCs at 1, 3, and 5 years are shown in Figure 8.

**Figure 7.**
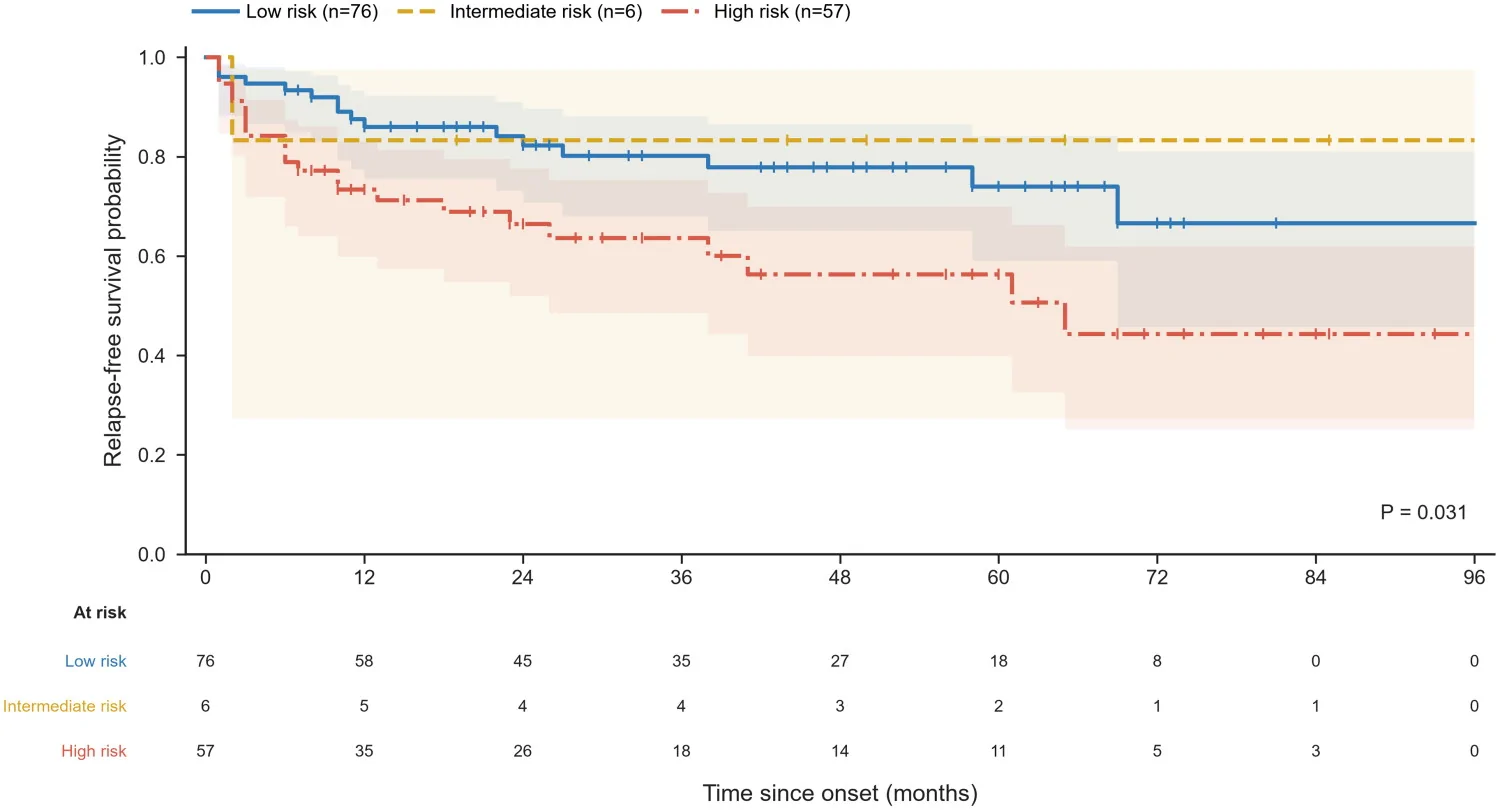
Kaplan–Meier curves for relapse-free survival stratified by pediatric phenotype risk group (exploratory model).

**Figure 8.**
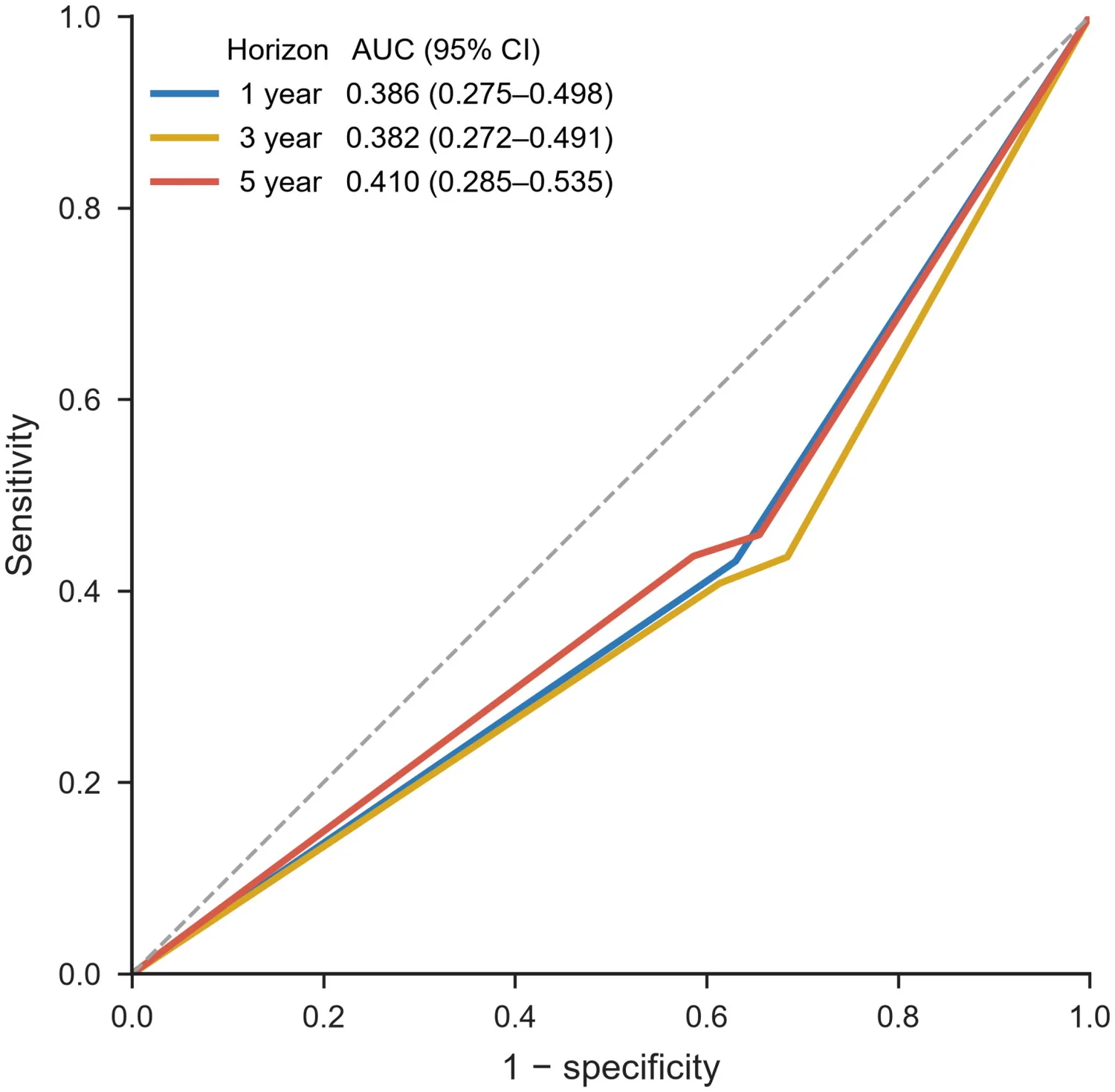
Exploratory pediatric phenotype model: time-dependent AUC of the phenotype-based risk model at 1, 3, and 5 years.

**Table 6.** One-year cumulative relapse risk by pediatric phenotype risk group (exploratory model).

Pediatric phenotype risk group	Total patients	Patients with relapse	1-year relapse rate, % (95% CI)
Low-risk	76	16	14 (5.5–21.6)
Intermediate-risk	6	1	16.7 (0–41.7)
High-risk	57	23	26.6 (14.1–37.3)

CI, confidence interval.

**Table 7.** Comparative performance metrics for the original MOG-AR score, the modified-threshold sensitivity analysis, and the exploratory pediatric phenotype model.

Model	C-index (95% CI)	3-year AUC (95% CI)	KM log-rank *p*
Original MOG-AR	0.472 (0.366–0.577)	0.494 (0.365–0.623)	0.032
Modified MOG-AR grade thresholds (sensitivity analysis)	0.472 (0.366–0.577)	0.494 (0.365–0.623)	0.3594
Pediatric phenotype model (exploratory)	0.394 (0.312–0.475)	0.382 (0.272–0.491)	0.0309

CI, confidence interval; AUC, area under the receiver operating characteristic curve; KM, Kaplan-Meier.

## Discussion

In this independent pediatric cohort, the MOG-AR score showed limited discrimination across all assessed time horizons and did not demonstrate a consistent gradient of first-relapse risk across grades. The predictor distribution was highly restricted, no individual score component was significantly associated with time to first relapse, and exploratory use of modified grade thresholds did not improve risk group separation. The phenotype-based model showed only a hypothesis-generating signal and poor discrimination.

Several features of the cohort restricted the effective score range. The age component contributed zero points in all 139 patients. Non-use of IST (95.0%) and OCS duration ≥3 months (96.4%) were near-constant treatment exposures rather than effective discriminators. Consequently, 94.3% of patients were concentrated in grades 2 and 3, limiting evaluation across the score’s intended predictor and risk range.

The overall log-rank test across the original MOG-AR grades was statistically significant. However, grade 4 included only two patients and had a highly imprecise 1-year relapse estimate of 50.0% (95% CI, 0.0–87.5%). The corresponding rates for grades 1, 2, and 3 were 16.7%, 14.9%, and 23.3%, respectively. These estimates did not demonstrate a consistent monotonic gradient; therefore, the overall *p* value should not be interpreted as evidence of clinically reliable risk stratification.

In the sensitivity analysis, the grade thresholds were shifted downward by two points while the continuous MOG-AR scores and patient rankings remained unchanged. The modified thresholds did not improve Kaplan–Meier separation (log-rank *p* = 0.359) or produce a meaningful gradient in 1-year relapse risk. Thus, alternative category cutoffs did not overcome the restricted predictor distribution in this cohort. This analysis represented an exploratory threshold reclassification rather than model updating.

MOG-AR performance has varied across external cohorts. Suboptimal discrimination was reported in Italian [5], North American [25], and UK cohorts [18], whereas an independent Chinese cohort reported adequate discrimination [17]. In our pediatric cohort, the noncontributory age component and restricted treatment exposures narrowed the effective score range and may have contributed to the limited discrimination observed. The sensitivity analysis further showed that simply modifying categorical thresholds did not improve risk group separation.

Importantly, the original MOG-AR score was derived using Andersen–Gill models within a recurrent-event framework, whereas the present analysis evaluated time from disease onset to first relapse. We applied the published scoring algorithm without modifying its components or point values but did not reproduce the original target outcome or recurrent-event model specification. The findings should therefore be interpreted as an external evaluation of the published score for first-relapse prediction in an independent pediatric cohort. Differences in outcome definition and case-mix may both have contributed to the lower performance observed in this study.

Treatment-related heterogeneity may partly contribute to differences in MOG-AR performance across cohorts. Post-attack IST was used in approximately 21.8% of the original cohort [10], 28.4% of the Italian cohort [5], 4.6% of the UK cohort [18], and 5.0% of our pediatric cohort. Because treatment-related variables contribute substantially to the MOG-AR score, these differences may influence score distributions and observed relapse risks. However, treatment practice alone is unlikely to explain the heterogeneous findings across external evaluations.

The exploratory phenotype-based model suggested that onset phenotype alone may carry a modest prognostic signal in children with MOGAD. The observed gradient across low-, intermediate-, and high-risk phenotype groups was directionally consistent with biological plausibility, as optic neuritis at onset has been consistently identified as a risk factor for a relapsing course in pediatric MOGAD [1], while ADEM presentation—particularly in younger children—has been associated with a predominantly monophasic course [20,21]. However, the intermediate-risk group was small (*n* = 6), limiting reliable estimation of event rates in that stratum. As this model was derived and evaluated within the same cohort, reported metrics reflect in-sample performance and are likely optimistic. These findings should be regarded strictly as hypothesis-generating; prospective construction and independent validation in adequately powered pediatric cohorts are required before any clinical application can be considered.

The limited predictive performance of the MOG-AR score in this cohort reflects the broader challenges in identifying reliable clinical predictors of pediatric MOGAD. Factors related to the relapse risk in children with MOGAD remain incompletely characterized, and findings across studies have been inconsistent [21]. The MOG-IgG antibody titer, one of the most clinically relevant prognostic parameters in MOGAD, was not included in the original model [10]. Persistent MOG-IgG positivity and high remission titers have been associated with an increased relapse risk, and seroconversion to negative titers has been associated with a marked reduction in the relapse incidence [4,12]. From an imaging perspective, new lesions identified on follow-up MRI are associated with subsequent relapses in pediatric MOGAD [26]. These observations suggest that longitudinal antibody dynamics and dynamic MRI features, rather than first-attack characteristics alone, may warrant inclusion in future pediatric-specific prediction models.

This study had several limitations. First, the age component contributed zero points in all patients, and the treatment-related components showed restricted variability. This case-mix limited evaluation across the score’s intended predictor and risk range. Second, the original MOG-AR score was derived in an Andersen–Gill recurrent-event framework, whereas this study evaluated time to first relapse and therefore did not reproduce the original outcome structure. Third, the single-center retrospective design may have introduced referral and information bias. Exact onset-to-treatment intervals were not consistently available, precluding reliable analysis of time to acute treatment. Fourth, only two patients were classified as grade 4, precluding reliable estimation in the highest-risk subgroup. Fifth, serial MOG-IgG titers were unavailable. Sixth, the cohort was recruited in central and southern China, which may limit generalizability to other populations. Finally, the exploratory pediatric phenotype model was derived and evaluated in the same cohort without external validation.

## Conclusions

In this independent, single-center pediatric MOGAD cohort, the published MOG-AR score did not provide clinically meaningful stratification of first-relapse risk. Restricted variability in the age and treatment-related components may have contributed to its limited performance. Further multicenter pediatric validation in pediatric cohorts with broader case-mix and treatment patterns is warranted before the score’s its general applicability in children can be established.

## Data Availability

The data that support the findings of this study are available on request from the corresponding author Sai Yang. The data are not publicly available due to restrictions imposed by the institutional ethics committee to protect the privacy of pediatric research participants.
